# The Association Between Dairy Product Consumption and Asthenozoospermia Risk: A Hospital-Based Case-Control Study

**DOI:** 10.3389/fnut.2021.714291

**Published:** 2021-10-21

**Authors:** Xiao-Bin Wang, Qi-Jun Wu, Fang-Hua Liu, Shuang Zhang, Han-Yuan Wang, Ren-Hao Guo, Xu Leng, Qiang Du, Yu-Hong Zhao, Bo-Chen Pan

**Affiliations:** ^1^Center for Reproductive Medicine, Shengjing Hospital of China Medical University, Shenyang, China; ^2^Department of Clinical Epidemiology, Shengjing Hospital of China Medical University, Shenyang, China; ^3^Clinical Research Center, Shengjing Hospital of China Medical University, Shenyang, China

**Keywords:** asthenozoospermia, case-control study, dairy product, nutrient, China

## Abstract

**Background:** Evidence of an association between dairy product and main related dairy nutrient intake, and the asthenozoospermia risk have been limited and controversial.

**Methods:** A hospital-based case-control study including 549 men with asthenozoospermia and 581 normozoospermic controls was carried out in the infertility clinics of Shengjing Hospital of China Medical University between June, 2020 and December, 2020. Dietary intake was assessed with a validated food frequency questionnaire. According to the World Health Organization guidelines, semen parameters were collected through masturbation and were measured with WLJY9000 instrument and flow cytometry. The daily intake of dairy products and related nutrients was categorized into three groups according to control distribution, and the lowest tertile was used as the reference category. An unconditional multiple logistic regression was used to estimate the odds ratios (ORs) and the corresponding 95% confidence intervals (CIs) for asthenozoospermia risk.

**Results:** After adjustment for potential confounders, we found no statistically significant associations between the intake of total dairy products and asthenozoospermia risk (OR_T3vs.T1_ = 1.19, 95%CI = 0.85–1.67). Additionally, we generated null findings regarding the main related nutrients from dairy, including protein (OR_T3vs.T1_ = 1.19, 95%CI = 0.85–1.68), fat (OR_T3vs.T1_ = 1.28, 95%CI = 0.91–1.80), calcium (OR_T3vs.T1_ = 1.20, 95%CI = 0.85–1.68), saturated fatty acids (OR_T3vs.T1_ = 1.30, 95%CI = 0.92–1.83), and phosphorous (OR_T3vs.T1_ = 1.18, 95%CI = 0.84–1.67), and the asthenozoospermia risk. Of note, after stratification by body mass index (BMI), and the saturated fatty acids consumption from dairy was significantly associated with a higher asthenozoospermia risk (OR_T3vs.T1_ = 1.76, 95%CI = 1.01–3.09) among participants with a BMI below 25 kg/m^2^.

**Conclusion:** This study provided limited evidence of an association between the intake of total dairy products and the main related dairy nutrients including protein, fat, calcium, saturated fatty acids, and phosphorus, and the asthenozoospermia risk. Further studies are warranted to confirm our findings in the future.

## Introduction

The decline in male reproductive health has raised serious concerns about the impact on human fertility (1). According to results from Global Burden of Disease 2017, the age-standardized prevalence of male infertility increased by 0.291% annually in 1990–2017 (2). Asthenozoospermia is a major pathological cause of male infertility. More than 40% of infertile men have this disease, which is linked to a reduction or lack of motile sperm in the ejaculate (3, 4). A recent report from China has shown that asthenozoospermia was present in 50.5% of 38,905 infertile male patients from 2008 to 2016 (5). Asthenozoospermia may be attributed to numerous causes, such as varicocele (6), genetic factors (7), infections (8), unhealthy lifestyle (9), environmental pollutants (10, 11), and lack of physical activity (12).

Recent evidence suggests that diet and nutrition might also play a role in the etiology of asthenozoospermia (13–16). Higher intake of sweets, processed meat, saturated fatty acids, and trans-fatty acids are associated with a higher risk of asthenozoospermia (15, 16). In contrast, higher intake of skim milk and dark green vegetables may reduce the risk of asthenozoospermia (13, 16). As a major part of people's daily diet, dairy products are rich in nutrients such as protein, fat, minerals, and vitamins, and are a major part of dietary recommendations worldwide (17). Well-known dairy products include whole milk, low fat milk, cheese, skim milk and yogurt (18). Dairy products are rich in protein, calcium, fat, saturated fatty acids, and phosphorus (19). However, up to now, research findings on the association between dairy products and male infertility are still inconsistent (16, 20–23). For example, a case-control study on 30 men with poor semen quality and 31 controls found a negative association between dairy product consumption and semen quality (22), but another case-control study including 72 asthenozoospermic men and 169 normozoospermic men did not show associations between dairy product intake and asthenozoospermia risk (16). We speculate that the inconsistent evidence might have been attribute to limited sample size and different adjustment for potential confounders in those studies. In addition, it would be necessary to explore the association between the main nutrients (protein, fat, calcium, saturated fatty acids, and phosphorus) in dairy products and asthenozoospermia risk as well as the total dairy products.

Whether dairy products intake in Chinese men is associated with asthenozoospermia risk remains an unresolved question. Therefore, through a hospital-based case-control study with a large sample size, we aimed to investigate the relationship between consumption of total dairy and their main nutrients including protein, calcium, fat, saturated fatty acids, and phosphorus and the risk of asthenozoospermia.

## Methods

### Design and Population

We performed a hospital-based case-control study in the infertility clinics at Shengjing Hospital of China Medical University, from June, 2020 to December, 2020. Incident cases (*n* = 597) were diagnosed with asthenozoospermia, defined by World Health Organization (WHO) guidelines (24) as a total motility (progressive + non-progressive) <40%, including both rapidly and slowly progressive motility, and non-progressive motility (24). Asthenozoospermia was also defined by progressive motility <32%, including rapidly and slowly progressive motility within 60 min of ejaculation over the prior 3 months (24). In defining asthenozoospermia, the total number (or concentration) of spermatozoa and the percentage of morphologically normal spermatozoa met or exceeded the lower reference limits. We excluded patients with a clinical history of varicocele. Eligible controls (*n* = 612) were normozoospermic men from infertile couples (more than 15 × 10^6^ of sperms/mL, more than 40% total motility, more than 32% progressive motility, and more than 4% normal form). Finally, 549 asthenozoospermia cases and 581 controls completed baseline survey and were included in the current analysis. The participation rates were 92% among cases and 95% among controls. The study protocol was approved by the ethics committee of Shengjing Hospital of China Medical University. Our study followed the “Strengthening the Reporting of Observational Studies in Epidemiology” guideline (Supplementary Table 1).

### Data Collection

The information collected in the questionnaire included general demographic characteristics, sleep condition, mental condition, history of health care product use and dietary consumption, personal lifestyle habits, physical activity, passive and indoor smoking, history of disease, and family history of chronic diseases. In addition, the height, weight, waist circumference, hip circumference, and blood pressure of participants were collected through physical examination.

### Semen Analyses

All the participants were asked to abstain from ejaculation for 3–7 days prior to the semen collection. Semen samples were collected in a sterilized container through masturbation in a dedicated semen collection room; condoms or lubricants were not used. Semen analysis was carried out soon after liquefaction (<60 min). Sperm motility was divided into three categories: progressive, including rapidly and slowly progressive, non-progressive and immotile according to the WHO criteria (24). Ejaculate volume and PH was directly measured, and additional parameters including sperm concentration, total sperm count, motility, and the percentage of each motility category of sperm were measured with WLJY9000, an instrument of computer-aided sperm analysis. Sperm DNA fragmentation and high sperm DNA stainability were assessed with flow cytometry. Papanicolaou staining of the semen smear was used, and the sperm morphology was determined through optical microscopy. Normal sperm reference values were determined according to WHO criteria (24). Throughout the study, external quality control was performed.

### Dietary Assessment

A validated semi-quantitative FFQ with 110 food items was used to assess the usual intake of food and nutrients. The FFQ in the current study was based on the FFQ used in a large, prospective, dynamic northeast cohort study in China (25). The reproducibility and validity of the questionnaire used in the current study were similar to questionnaire used in northeast cohort study in China. Men were asked to indicate how often, on average, they had consumed each type of food in the year before diagnosis. The FFQ included seven options for how often each food was consumed, including “more than two times per day,” “1–2 times per day,” “4–6 times per week,” “2–3 times per week,” “one time per week,” “2–3 times per month,” or “never.” The nutrients in each food of FFQ were calculated according to The Chinese Food Composition Tables (26). Total dairy intake was calculated by summing up intake amounts of whole milk, low-fat dairy, yogurt, and cheese. The auditor reviewed all FFQ options, and any missing or unclear options were discussed with the respondent and corrected over time.

### Statistical Analysis

The Kolmogorov–Smirnov statistic was used to test the normality of all continuous variables. Student's *t*-tests were used to compare continuous variables between groups, whereas the chi-square test was used to compare categorical variables. The results are presented as means with standard deviation (SD) for continuous variables and as frequency with percentage for categorical variables. The daily intake of dairy products and their nutrients was categorized into three groups, and the lowest tertile in the controls was used as the reference category. When these variables were treated as continuous variables, the efficiency slope was one SD according to the intake of controls. An unconditional multiple logistic regression model was used to estimate odds ratios (ORs) and the corresponding 95% confidence intervals (CIs) for the association of dairy products and their main nutrients intake and the asthenozoospermia risk. Model 1 adjusted for age (years). Model 2 was adjusted for age (years) as well as BMI (kg/m^2^), smoking status (no/yes), household income (RMB thousand yuan), education level (junior secondary or below, senior high school/technical secondary school, and junior college/university or above), physical activity (MET/hours/days), occupation (worker, office staff, professional technicians, enterprise staff, freelancer, other occupations), total energy intake (kcal), and abstinence time (days). Moreover, in order to reduce the confounding effect of dietary factors, we further adjusted dietary pattern in our analysis. Dietary pattern, which indicated optimal uncorrelated dietary factors, had a simpler structure with improved interpretability (14). We used principal components method for factor analysis to derive potential dietary patterns in the present study (13). There was no collinearity among all the variables in this analysis. We also calculated the adjusted risk estimates for asthenozoospermia through intake of dairy products and their main nutrients (protein, fat, calcium, saturated fatty acids, and phosphorus), stratified by BMI. All analyses were performed with SAS version 9.4 (SAS Institute Inc., Cary, NC, USA). Statistical significance was set at *p* < 0.05 and was based on a two-sided test.

## Results

The distribution of general characteristics among cases and controls is presented in Table 1. The mean age and abstinence time for cases were significantly higher than those for controls. Cases had significantly lower sperm concentration, total sperm count, progressive motility, total motility, and percentage of normal sperm morphology than controls. In terms of dietary factors, the mean egg intake in cases was significantly higher than that in controls, whereas the mean meat intake in cases was significantly lower than that in controls.

**Table 1:** General characteristics of the participants.

**Characteristics**	**Asthenozoospermia**	**Normal**	***P*** **value**
No. of participants	549	581	
Age (years)	33.29 ± 5.28	32.11 ± 4.50	<0.05
Body mass index (kg/m^2^)	26.41 ± 4.42	26.25 ± 4.55	0.55
Physical activity (MET/h/week)	166.90 ± 103.40	165.90 ± 101.80	0.87
Television watching (h/week)	6.52 ± 8.79	6.19 ± 8.01	0.52
Computer using (h/week)	24.81 ± 15.95	24.90 ± 15.07	0.93
Abstinence time (days)	4.47 ± 1.48	4.28 ± 1.39	<0.05
**Semen parameters**			
Ejaculate volume (ml)	3.61 ± 1.47	3.45 ± 1.26	0.05
Sperm concentration (10^6^/ml)	58.74 ± 36.13	71.10 ± 39.88	<0.05
Total sperm count (10^6^/ml)	198.80 ± 126.00	232.20 ± 133.50	<0.05
Progressive motility (%)	22.02 ± 8.72	44.58 ± 9.34	<0.05
Total motility (%)	27.94 ± 10.92	54.94 ± 11.35	<0.05
Normal sperm morphology (%)	5.70 ± 2.55	6.68 ± 2.73	<0.05
**Diet**			
Meat (g/day)	99.81 ± 45.88	106.70 ± 49.02	<0.05
Eggs (g/day)	36.03 ± 27.91	32.22 ± 25.26	<0.05
Fish and seafood (g/day)	22.94 ± 19.69	22.84 ± 20.49	0.93
Beans and bean products (g/day)	97.86 ± 83.31	92.10 ± 82.43	0.24
Vegetables (g/day)	206.10 ± 142.00	194.00 ± 130.20	0.14
Fruits (g/day)	173.50 ± 194.50	167.20 ± 171.60	0.56
Total energy intake (kcal/day)	1,822.70 ± 579.90	1,769.10 ± 562.00	0.12
Smoking status (*n*, %)			0.11
No	285 (51.91)	274 (47.16)	
Yes	264 (48.09)	307 (52.84)	
Educational level (*n*, %)			0.67
Junior secondary or below	121 (22.04)	141 (24.27)	
Senior high school/technical secondary school	79 (14.39)	82 (14.11)	
Junior college/university or above	349 (63.57)	358 (61.62)	
Annual family income (RMB thousand yuan) (*n*, %)			0.76
<50	98 (17.85)	94 (16.18)	
50 to <100	209 (38.07)	226 (38.90)	
≥100	242 (44.08)	261 (44.92)	

*MET, metabolic equivalent. All continuous variables are shown as the mean and standard deviation. P values were determined with Student's t-test or chi-square test. All statistical tests are two sided*.

Table 2 shows the estimated adjusted ORs and 95%CIs resulting from the multivariable logistic regression models regarding the association between tertiles of dairy product and related dairy nutrients intake and asthenozoospermia. Overall, we found no statistically significant associations between the intake of total dairy products and the asthenozoospermia risk in the age-adjusted or multivariable-adjusted models. Additionally, when we focused on the main nutrients in dairy products, we failed to detect any significant associations between protein, fat, calcium, saturated fatty acids, and phosphorus intake and the risk of asthenozoospermia. Furthermore, similar results were also seen for total dairy products, protein, fat, calcium, saturated fatty acids, and phosphorus intake when they were treated as continuous variables for one SD increment.

**Table 2:** Adjusted ORs and 95%CIs for asthenozoospermia by intake of dairy products and related nutrients.

**Variables**	**Cases (*N* = 549)**	**Controls (*N* = 581)**	**Model 1^b^**	**Model 2^c^**
**Total dairy**^a^ (g/day)				
T1 (<31.16)	149	176	1.00 (Ref)	1.00 (Ref)
T2 (31.16 to <135.66)	193	197	1.19 (0.89–1.61)	1.20 (0.88–1.63)
T3 (≥135.66)	207	208	1.21 (0.90–1.62)	1.14 (0.82–1.58)
*P* for trend			0.33	0.64
Per 1-SD increment			0.91 (0.81–1.02)	0.91 (0.79–1.04)
**Protein from dairy (g/day)**				
T1 (<0.95)	147	173	1.00 (Ref)	1.00 (Ref)
T2 (0.95 to <4.16)	201	207	1.18 (0.87–1.58)	1.18 (0.87–1.60)
T3 (≥4.16)	201	201	1.20 (0.90–1.62)	1.14 (0.82–1.59)
*P* for trend			0.32	0.62
Per 1-SD increment			0.91 (0.81–1.02)	0.91 (0.79–1.04)
**Fat from dairy (g/day)**				
T1 (<0.97)	146	180	1.00 (Ref)	1.00 (Ref)
T2 (0.97 to <3.64)	202	202	1.28 (0.95–1.73)	1.30 (0.96–1.76)
T3 (≥3.64)	201	199	1.28 (0.95–1.72)	1.23 (0.88–1.71)
*P* for trend			0.25	0.50
Per 1-SD increment			0.92 (0.82–1.03)	0.92 (0.80–1.06)
**Calcium from dairy (mg/day)**				
T1 (<36.44)	150	180	1.00 (Ref)	1.00 (Ref)
T2 (36.44 to <158.65)	201	200	1.24 (0.93–1.67)	1.25 (0.92–1.70)
T3 (≥158.65)	198	201	1.22 (0.91–1.64)	1.15 (0.83–1.60)
*P* for trend			0.33	0.66
Per 1-SD increment			0.91 (0.81–1.02)	0.91 (0.79–1.04)
**Saturated fatty acids from dairy (g/day)**				
T1 (<0.58)	146	180	1.00 (Ref)	1.00 (Ref)
T2 (0.58 to <2.21)	200	202	1.27 (0.94–1.71)	1.33 (0.98–1.82)
T3 (≥2.21)	203	199	1.29 (0.96–1.74)	1.30 (0.92–1.83)
*P* for trend			0.20	0.36
Per 1-SD increment			0.92 (0.82–1.03)	0.92 (0.80–1.05)
**Phosphorus from dairy (mg/day)**				
T1 (<25.98)	147	173	1.00 (Ref)	1.00 (Ref)
T2 (25.98 to <113.10)	202	208	1.18 (0.88–1.58)	1.23 (0.90–1.68)
T3 (≥113.10)	200	200	1.20 (0.89–1.62)	1.18 (0.84–1.67)
*P* for trend			0.32	0.55
Per 1-SD increment			0.91 (0.81–1.01)	0.90 (0.79–1.04)

*CI, confidence interval; OR, odds ratio; T, tertiles; Ref, reference*.

a *Total dairy includes whole milk, skim/low-fat milk, cheese, and yogurt*.

b *Model 1: adjusted for age*.

c *Model 2: adjusted for age, body mass index, smoking status, household income, education level, physical activity, occupation, total energy intake, abstinence time, and dietary patterns except those for dairy products*.

**Figure 1:**
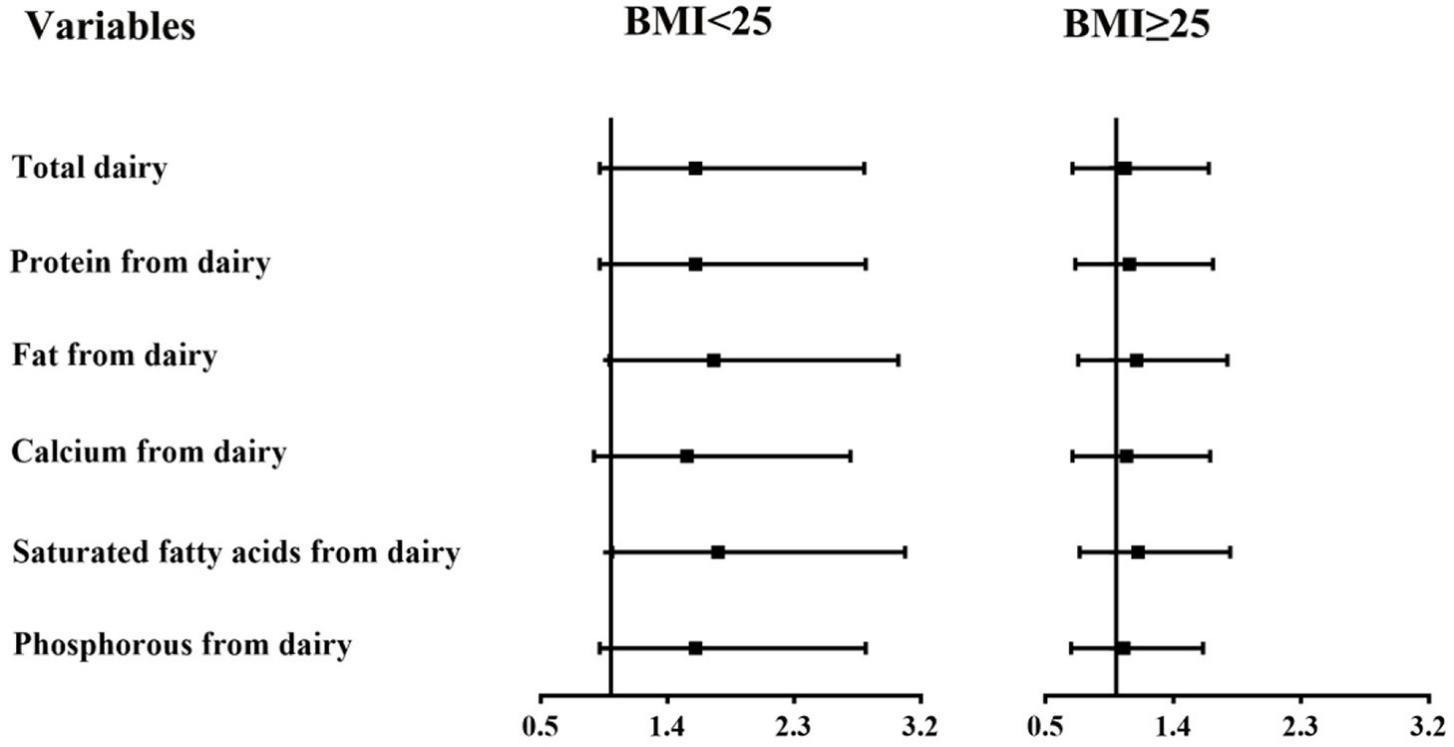
The associations between intake of dairy products and related nutrients and the risk of asthenozoospermia in strata by body mass index (BMI) (<25 and ≥25 kg/m^2^). The first tertile was used as a reference, and the third tertile was compared with the reference to calculate the effect estimates. The logistic regression model was adjusted for age, smoking status, household income, education level, physical activity, occupation, total energy intake, abstinence time, and dietary patterns except those for dairy products. *p*-interaction between intake of dairy products and related nutrients and BMI was calculated. All *p*-interaction were >0.05.

**Figure 2:**
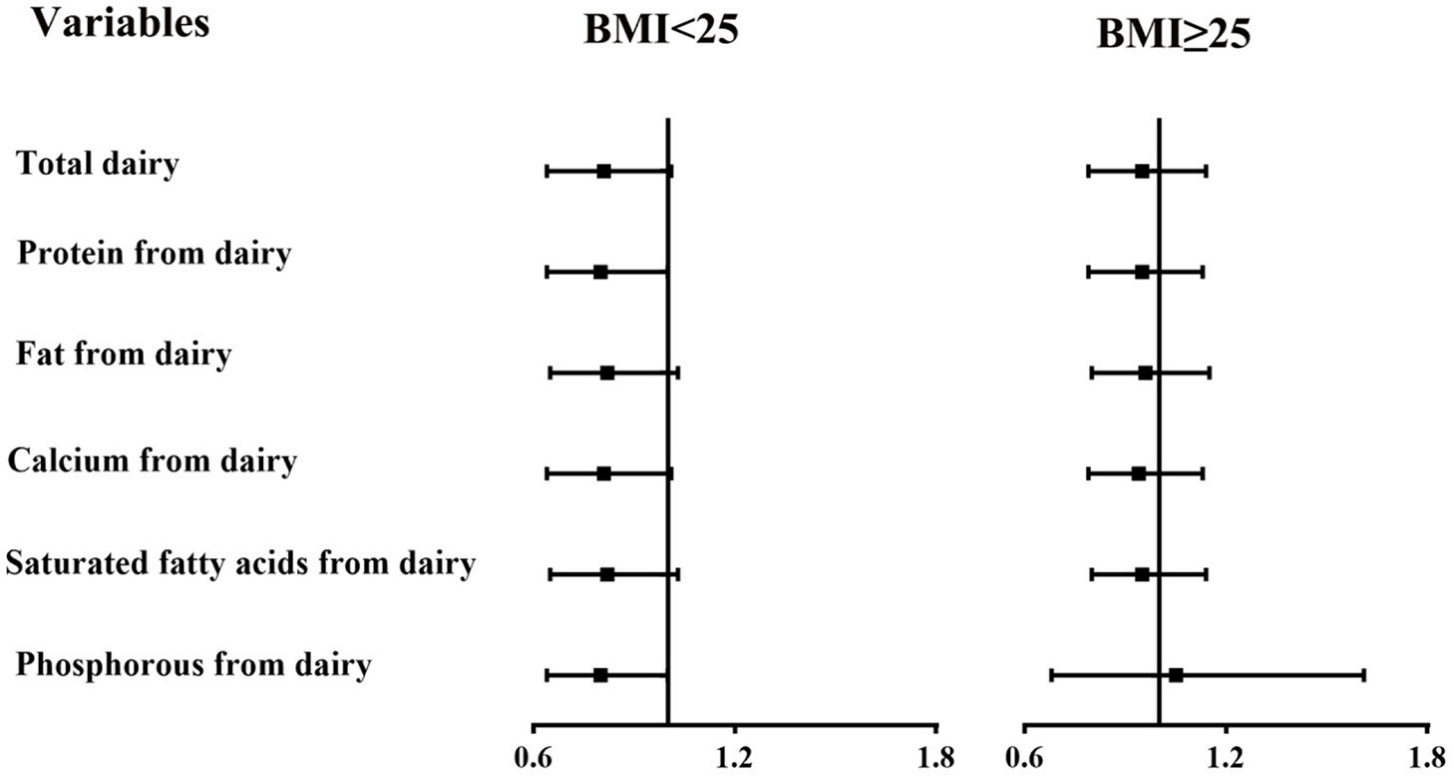
The associations between intake of dairy products and related nutrients and the risk of asthenozoospermia in strata by body mass index (BMI) (<25 and ≥25 kg/m^2^). The efficiency slope for all variables was one standard deviation. The logistic regression model was adjusted for age, smoking status, household income, education level, physical activity, occupation, total energy intake, abstinence time, and dietary patterns except those for dairy products.

In subgroup analyses stratified by BMI (Figures 1, 2), the participants with a BMI below 25 kg/m^2^ in the highest tertile of saturated fatty acids from dairy intake showed an association with a higher risk of asthenozoospermia (OR = 1.76, 95% CI = 1.01–3.09). However, other related nutrients from dairy intake showed no statistically significant associations with the risk of asthenozoospermia in participants with a BMI <25 or >25 kg/m^2^.

## Discussion

In this hospital-based case-control study, we did not find any evidence of an association between intake of total dairy products or their main nutrients, and the risk of asthenozoospermia. However, subgroup analyses showed that the consumption of saturated fatty acids from dairy products was associated with increased risk of asthenozoospermia among participants with a BMI below 25 kg/m^2^.

Our findings are consistent with those from several existing studies on dairy products and male infertility (15, 16, 20, 23) (Table 3). For example, a case-control study with 72 asthenozoospermic men and 169 normozoospermic men explored the association of intake of different food groups with asthenozoospermia in Iran, and found that there was no change in the odds of asthenozoospermia with increasing total dairy product intake (16). Similarly, Vujkovic et al. (23) performed a cross-sectional study with 161 men in subfertile couples in Spain, and they did not find any correlation between the intake of dairy products and sperm parameters. In addition, a longitudinal study in the United States observed a similar result (20). Finally, Eslamian et al. (15) reported that being in the highest tertile of total saturated fatty acids was positively associated with asthenozoospermia among 107 men with incident asthenozoospermia and 235 age-matched controls. In contrast, our results did not agree with some prior findings (21, 22) (Table 3). In a case-control study recruiting 31 patients with normospermia and 30 patients with oligoasthenoteratospermia in a fertility clinic, Mendiola et al. found that the control group had a lower intake of total dairy products (semi-skimmed milk, cheese, whole milk, and yogurt) than the case group (22); this discrepancy might be attributable to a small sample size. Additionally, a cross-sectional study of 189 young men in the USA suggested that the intake of total dairy products was negatively correlated with sperm morphology, and this relationship was mainly caused by whole-fat dairy product intake (21). However, the age of the population in this study (mean = 19.48 years) was younger than that in our study (mean = 32.68 years). Differences in dairy intake by regions may also account for the inconsistent results. Compared with an average dairy products intake of ~118.22 g/d in our study, the mean intake of dairy products in the United States was much higher, ~268.8 g/day (27). It is important to note that smokers have accounted for nearly 50% of all participants, this percent is close to a previous study from the China Kadoorie Biobank project, smokers aged between 30 and 69 accounted for more than 50% (28).

**Table 3:** Characteristics of studies examining the association between dairy products and asthenozoospermia.

**References, country**	**Study design**	**Study population (age)**	**Dietary assessment**	**Exposure factors**	**Outcome**	**OR (95%CI)**	**Adjustments**
Afeiche et al. (21), America	Cross-sectional study	Men: 189 (18~22 years)	FFQ (131, past year)	Total dairy foodFull-fat dairy food^a^	Total sperm Count (million) Sperm concentration (million/ml) Progressive motility (% motile) Sperm morphology (% normal) Ejaculate volume (ml)	N.R.	Age, abstinence time, race, smoking status, BMI, recruitment period, moderate-to-intense exercise, TV watching, alcohol intake, prudent and western dietary patterns, and total calorie intake
Eslamian et al. (16), Iran	Case-control study	Cases: 72 Controls: 169 (20~40 years)	FFQ (168, past year)	Dairy products	Asthenozoospermia	OR: 2.91 (0.88–3.03)	Age, BMI, total energy intake, smoking status and heavy traffic near home, and specific food groups
Mendiola et al. (22), Spain	Case-control study	Cases: 30	FFQ (96, past year)	Dairy products	Poor semen quality	OR: 3.1 (1.1–8.5)	N.R.
		Controls: 31 (mean age: 33.49 years)					
Afeiche et al. (20), America	Longitudinal study	Men: 155 (18~55y)	FFQ (131, past year)	Full-fat dairy food^b^Whole milk	Total sperm count (million) Sperm concentration (million/ml) Progressive motility (% motile) Sperm morphology (% normal) Ejaculate volume (ml)	N.R.	Age, total energy intake, body mass index, smoking status, abstinence time, previous infertility diagnosis, race, and dietary patterns
Vujkovic et al. (23), The Netherlands	Prospective study	Men: 161 (18~55 years)	FFQ (195, past 4 weeks)	Dairy products	DFI (%)	N.R.	Age, BMI, smoking, and vitamin supplement use
					Volume (ml)		
					Concentration (× 10^6^ cell/ml)		
					Motility (%)		
					Morphology (%)		

*FFQ, food frequency questionnaire; BMI, body mass index; OR, odds ratio; DFI, DNA fragmentation index; N.R., not reported*.

a *Includes whole milk, cream, ice cream, cream cheese, and other cheese*.

b *Includes cheese, cream, ice cream, and whole milk*.

Several reports have suggested the potential biological mechanisms between dairy intake and asthenozoospermia risk. Milk obtained from pregnant cows contains natural estrogens (29). Men who consume this kind of milk show significantly increased serum estrone and progesterone concentrations, and decreased serum luteinizing hormone, follicle-stimulating hormone, and testosterone concentrations, which can decrease sperm production (30). In addition, asthenozoospermic men have higher saturated fatty acid concentrations in their sperm membrane compared with normozoospermic men (31). Dietary intake of saturated fatty acids may affect sperm quality by affecting fatty acids profiling (32).

Several studies have indicated that high BMI may decrease sperm quality (33, 34). Differentially expressed proteins are mainly associated with protein degradation involved in spermiogenesis and sperm motility, and these differences may play critical roles in obesity-associated asthenozoospermia (34). Moreover, a study showed that unit increases in BMI compared to the nonobese were inversely associated with sperm motility (33). Interestingly, after stratification by BMI, we observed that a higher intake of saturated fatty acids from dairy products increased the risk of asthenozoospermia among participants with a BMI below 25 kg/m^2^. Furthermore, the point estimates of these findings slightly differed (1.76 vs. 1.01 for the highest tertile). Because we were unable to rule out the possibility of spurious findings, further studies are warranted to validate our results.

Several advantages are worth mentioning. First, the consumption of dairy products and their nutrients was estimated with a validated FFQ representing the general population in northeast China. Second, the present study included a relatively large sample size of cases (*n* = 549) and controls (*n* = 581), thus enabling us to generate more reliable results. The high participation rate (92% among cases and 95% among controls) in our study was also an important advantage. Third, the study adjusted for a relatively large number of confounding factors, such as age, BMI, smoking, education level, total energy, and dietary patterns, thus adding to the credibility of the conclusions.

However, several limitations also must be noted. First, due to recall or selection bias which was inherent in the case-control study, causality is unlikely to be determined from a temporal perspective. Additionally, hospital-based case-control studies inherently cannot avoid selection and recall bias. In a hospital-based case-control study, patients might have recalled different eating habits after being diagnosed with asthenozoospermia. Additionally, the cases did not comprise a random sample of all patients, and the controls came from normozoospermic men in infertile couples, instead of a random sample of the general population, thus potentially leading to admission rate bias. However, we recruited the controls in the same clinical setting (the infertility clinic), and well-trained investigators collected the patients' dietary information with a validated FFQ, thus potentially reducing recall bias and increasing the comparability of case and control information (35, 36). Second, the FFQ was used to evaluate dietary intake in these participants. The standard food portions were applied to calculate food intake, but the intake of participants could not be accurately calculated, thus potentially resulting in an underestimation of the relationship between exposure and outcome. However, we used a validated FFQ and excluded participants with higher energy intake (>4,200 kcal) and lower energy intake (<800 kcal). Third, owing to the low intake of various dairy products, we were unable to assess the relationship between various dairy products (including whole milk, skim/low fat milk, cheese, and yogurt) and asthenozoospermia. Fourth, our study might not have completely excluded unmeasured or residual confounding variables, such as air pollution (PM_10_, SO_2_, and NO_2_) (37) or genetic factors (38), thus possibly affecting the aforementioned association. Further studies are needed to exclude these potential problems and to better elucidate the relationship between dairy product consumption and asthenozoospermia.

In conclusion, the present hospital-based case-control study suggested no statistically significant associations between the intake of total dairy products or their main nutrients, and the risk of asthenozoospermia. Of note, subgroup analyses indicated that when the BMI was below 25 kg/m^2^, higher intake of saturated fatty acids from dairy was associated with an increased risk of asthenozoospermia. Our findings may aid in developing guidance for dairy product intake for patients with clinical asthenozoospermia. Future large prospective studies are needed to confirm our findings. Further studies should be conducted to explore the association of the total nutrients content of the diet with the risk of asthenozoospermia.

## Data Availability Statement

The datasets presented in this article are not readily available because of ethical, legal, and privacy issues. Requests to access the datasets should be directed to Corresponding author. Requests to access these datasets should be directed to B-CP, panbochen@cmu.edu.cn.

## Ethics Statement

The studies involving human participants were reviewed and approved by Shengjing Hospital of China Medical University. The patients/participants provided their written informed consent to participate in this study.

## Author Contributions

B-CP and Y-HZ: conception and design of the study. X-BW: draft the article. R-HG, XL, and QD: collect data. Q-JW and SZ: analysis and interpret data. F-HL and H-YW: revise the article critically for important intellectual content. All authors read and approved the final manuscript.

## Funding

This research was funded by the National Key R&D Program of China (No. 2017YFC0907403 to Y-HZ) and Shengjing Hospital Clinical Research Project (No. M0071 to B-CP).

## Conflict of Interest

The authors declare that the research was conducted in the absence of any commercial or financial relationships that could be construed as a potential conflict of interest.

## Publisher's Note

All claims expressed in this article are solely those of the authors and do not necessarily represent those of their affiliated organizations, or those of the publisher, the editors and the reviewers. Any product that may be evaluated in this article, or claim that may be made by its manufacturer, is not guaranteed or endorsed by the publisher.
